# Biofilm Production in Intensive Care Units: Challenges and Implications

**DOI:** 10.3390/pathogens13110954

**Published:** 2024-11-01

**Authors:** Ferdiye Taner, Buket Baddal, Liana Theodoridis, Steve Petrovski

**Affiliations:** 1 Department of Medical Microbiology and Clinical Microbiology, Faculty of Medicine, Near East University, 99138 Nicosia, Cyprus; buket.baddal@neu.edu.tr; 2 DESAM Research Institute, Near East University, 99138 Nicosia, Cyprus; 3 Department of Physiology, Anatomy, and Microbiology, La Trobe University, Bundoora, VIC 3086, Australia; liana.theodoridis@latrobe.edu.au (L.T.); steve.petrovski@latrobe.edu.au (S.P.)

**Keywords:** ICU, bacterial biofilm production, hospital-acquired infection, antibiotic resistance, indwelling medical devices, molecular genetics

## Abstract

The prevalence of infections amongst intensive care unit (ICU) patients is inevitably high, and the ICU is considered the epicenter for the spread of multidrug-resistant bacteria. Multiple studies have focused on the microbial diversity largely inhabiting ICUs that continues to flourish despite treatment with various antibiotics, investigating the factors that influence the spread of these pathogens, with the aim of implementing sufficient monitoring and infection control methods. Despite joint efforts from healthcare providers and policymakers, ICUs remain a hub for healthcare-associated infections. While persistence is a unique strategy used by these pathogens, multiple other factors can lead to persistent infections and antimicrobial tolerance in the ICU. Despite the recognition of the detrimental effects biofilm-producing pathogens have on ICU patients, overcoming biofilm formation in ICUs continues to be a challenge. This review focuses on various facets of ICUs that may contribute to and/or enhance biofilm production. A comprehensive survey of the literature reveals the apparent need for additional molecular studies to assist in understanding the relationship between biofilm regulation and the adaptive behavior of pathogens in the ICU environment. A better understanding of the interplay between biofilm production and antibiotic resistance within the environmental cues exhibited particularly by the ICU may also reveal ways to limit biofilm production and indivertibly control the spread of antibiotic-resistant pathogens in ICUs.

## 1. Introduction

Intensive care unit (ICU)-acquired infections continue to cause considerable morbidity, and the treatment of these infections is increasingly problematic [1,2,3]. Continuing to dominate the causes of persisting infections are the “ESKAPE” organisms (*Enterococcus faecium*, *Staphylococcus aureus*, *Klebsiella pneumoniae*, *Acinetobacter baumannii*, *Pseudomonas aeruginosa*, and *Enterobacter* spp.) [4,5]. As these bacteria are associated with resistance to multiple antibiotics and at times are also found residing in biofilms, the “perfect storm” is created and the crisis escalates. Scientists have recognized resistance throughout the antibiotic era, and it has been shown that biofilms increase drug resistance by delayed penetration and altered growth rate [6,7]. However, with the current understanding of antibiotic resistance and biofilm production, is the health sector any closer to managing and treating ICU-related infections? Will the slowdown in the availability of new antibiotic therapies and the challenges presented by biofilm production continue to hinder our abilities in the clinical management of ICU-related infections? Are there additional environmental factors such as device design and clinical surfaces that will always favor biofilm-producing microorganisms? Has the rapid explosion in the availability of genome sequences added to our understanding of biofilm production in ICU-related infections? Will the health sector ever be at the forefront of managing antibiotic resistance and biofilm production in ICUs?

To unravel and discuss some of these questions put forward, this paper will begin with a brief overview of the prevalence of ICU-related biofilm production and the relationship shared with antibiotic resistance. It will then establish the advances made in the understanding of molecular genetics among ICU bacteria associated with biofilm production and antibiotic resistance. This paper will also provide insights into how bacteriophage therapy and artificial intelligence may assist in treatment.

## 2. The ICU—A Hub for Healthcare-Associated Infections (HAIs)

Healthcare-associated infections (HAIs) represent the most common unfavorable event to occur among hospitalized patients [8,9]. Recent statistics suggest that 1 in 10 affected patients will die from an HAI; the most common of these include pneumonia, surgical site infections, and bloodstream infections [8,9,10]. The duration of hospitalization and the type of HAI can significantly increase mortality and hospital-associated costs, particularly if the isolates causing the HAI are multidrug-resistant (MDR) [11,12,13,14]. Considering that the ICU provides greater medical attention to patients who are critically ill, the unintended increased risk for infection by grouping critically ill patients together continues to be a drawback.

Distinguishing whether an infection was acquired prior to ICU admission can at times pose a challenge. For consistency across international health surveillance reports, infections acquired 48 h after admission to the ICU are considered to be ICU-acquired, with day 1 representing the day of admission into the ICU. The onset of infection from day 3 onward following admission into the ICU should be reported as an ICU infection [12,15]. Patients in the ICU are also exposed to mechanical and device interventions, such as ventilators, central venous catheters, and urinary tract catheters [3,16]. Therefore, HAIs are obtained at a higher rate, primarily due to the more invasive procedures and device-dependent treatment protocols incorporated to treat patients [11,12]. Of all HAIs, one-quarter occur in ICUs [17]. Subsequently, the most common clinically significant infections observed in the ICU are ventilator-associated pneumonia (VAP), catheter-associated urinary tract infections (CA-UTIs), and intravascular catheter-related bloodstream infections (CR-BSIs) [9,18,19]. These infections are invariably associated with an increased length of stay in the ICU, underlying conditions of the patient, the cause for admission, and patient age [20]. Despite the various types of ICUs present in hospitals, for example, the pediatric ICU, the trauma ICU, and the neonatal ICU, data regarding differences in HAIs across these various ICUs are limited. One particular study did, however, focus on profiling microbial communities located on the surfaces of the neonatal ICU [8]. Furthermore, hospitals’ healthcare processes and climatological conditions can all contribute to the complications related to the prevalence of ICU-related HIAs [15] and all warrant in-depth discussion.

The purpose of this review, however, is to highlight and explore the complex interplay of pathogen virulence factors, namely biofilm production, the association it may have with antibiotic resistance, and the continued burden these two virulence factors are causing in ICUs.

## 3. An ICU Perspective on Biofilm-Associated Bacteria

### 3.1. Biofilm Formation on Non-Medical Device Surfaces

The initial contamination of inanimate surfaces in the ICU may occur by either transfer from health workers’ hands or direct patient shedding of bacteria in the immediate environment of the patient [21]. It is estimated that 40–60% of ICU-related HAIs are linked to the normal flora of patients, while 20–40% are a consequence of cross-infection from healthcare workers [22,23]. In addition, the frequent handling and contact with medical devices, workstations, keyboards, and medical records contribute to a widespread contamination issue [21,24,25,26]. Bacterial cultures isolated from ICU surfaces can include *Enterococcus faecalis*, *Staphylococcus aureus*, *Pseudomonas aeruginosa*, *Klebsiella pneumoniae*, and *Acinetobacter baumannii* [26]. This observation coincides with reports from the EU, West Asia, and Africa, where *P. aeruginosa*, *K. pneumoniae,* and *A. baumannii* are the most common causes of ICU-related infections [4,11,12,23]. In one cohort study, *A. baumannii* was isolated from one-third of gowns and gloves of health workers following the treatment of a patient infected with *A. baumannii*, while other studies have cultured *S. aureus* from Venetian blind cords, mattress bay, and wash basin rubbers [27]. Viable bacteria have also been sampled from surfaces and furnishings despite the terminal cleaning of an ICU ward [5], demonstrating the possibility of health care workers contaminating their hands after contact with inanimate surfaces despite following hygiene protocols prior to entering the ICU. A randomized cross-over study demonstrated that following a period of 4 h after standard cleaning measures were applied, high-contact surfaces were re-contaminated [27]. These examples form part of a collection of studies that demonstrate that pathogens persist on surfaces despite terminal cleaning [5,26]. The implication of these studies is that ICU-acquired contamination has the potential to cross-contaminate new ICU patients and raises the possibility of the ICU being a reservoir for persistent pathogens. A prospective cohort study revealed that risk of ICU patients acquiring *P. aeruginosa* and *A. baumannii* from prior room patient occupants is severe [26]. The presence and persistence of pathogens on ICU dry surfaces can be related to environmental factors such as the temperature and humidity of the room and the concentration of organic substances present on surfaces [28]. The unique characteristics of pathogens can also contribute to their viability on surfaces; biofilm formation is a property hat appears to largely contribute to pathogen persistence on ICU surfaces [5,26,29].

Biofilms by definition are sessile polymicrobial communities that adhere to biotic and abiotic surfaces and are encased within a self-produced extracellular polymeric matrix [30,31]. Bacteria use biofilm production as part of their adaptive and survival mechanisms; therefore, a variety of bacterial species are capable of producing biofilms and thus are ubiquitous in nature [17,30,31]. The matrix-encased community of bacteria forms aggregates that exhibit a complex network of water channels, which permit the flow of nutrients and oxygen, allowing bacteria to persist and survive even in the most nutrient-deprived environments [31]. The detection of biofilm-forming bacteria in various niches (e.g., water system piping, natural aquatic systems, and plant roots) was a trigger for the pursuit and recognition of microbial biofilms involved in pathogenesis and disease [17,30]. Considering the protective armor and adhesion properties biofilm production provides to bacteria, it is not surprising that a spectrum of dry healthcare surfaces (>90%) and indwelling medical devices in the ICU have been shown to harbor biofilms [5,30,32]. Using scanning electron microscopy, Vickery et al. were able to demonstrate that sampled ICU furnishing surfaces (e.g., Venetian blind cord and sterile supply bucket) were contaminated with *S. aureus* embedded in biofilm following terminal cleaning [27].

The complications associated with biofilm-associated bacteria include the difficulty of its removal from contaminated surfaces. Consequently, dry surface biofilms have been shown to persist for long periods of time and on most highly touched surfaces in the ICU. In one study, among 57 ICU surfaces, microscopy analysis revealed that biofilms were present on all surfaces [29]. It is a concern that biofilm-contaminated surfaces remained unchanged in their total number, whether or not surfaces were cleaned twice a day [29]. Additionally, comparative studies between biofilm-encased bacteria with planktonic bacteria growing in liquid culture are 100–250 times more resistant to biocides [22]. Evidently, biofilm production promotes the persistence of bacteria following the disinfection of inanimate dry surfaces. This highlights that cleaning practices may be suboptimal, with both detergents and disinfectants unable to sufficiently remove biofilm-encased bacteria in comparison to planktonic cells. The resilience biofilms provides a structured community of microbial cells against physical removal, and chemical disinfection is largely due to the presence of extracellular polysaccharide (EPS), which is distributed between cells in a non-homogeneous manner [5,33,34]. EPS encompasses the microbial cells to form a matrix, up to 90% of a biofilm may be accounted for by an EPS [32]. EPSs, together with the presence of fimbriae, flagella, and cell surface hydrophobicity, influence the extent and rate of the attachment of cells to surfaces [30,34].

The available literature related to the direct correlation between biofilm-contaminated surfaces in the ICU and transmissibility to patients is limited and warrants further investigation. In addition, it becomes evident that the intent of most studies related to ICU surface contamination is to investigate the epidemiology and to identify reservoirs of potentially pathogenic bacteria prior to and after the cleaning of the ICU [3,5,8,19,21,24]. Although the persistence of bacteria on dry surfaces is related to biofilm production, the overall composition and architecture of biofilms across these studies are not investigated in great detail and require a different research approach. This is an area of study that requires further attention in order to gauge whether the composition/architecture of biofilms is unique across hospitals and how different hospital cleaning protocols alter the persistence of biofilm-associated bacteria on dry surfaces. Such studies may reveal a correlation between disinfection regimens, biofilm “type”, and persistence.

### 3.2. VAP Associated Biofilm Formation in the ICU

Over the past decade, research into medical-device-related infections has become prominent. Dominating this area of research is ventilator-associated pneumonia (VAP) [35,36]. While some studies focus on the management of VAP in the ICU, in other studies that investigate the pathogenesis of VAP, the immediate trend that becomes apparent is the incidence of biofilm-forming pathogens that colonize the inner endotracheal tube (ET) [35,36,37]. Although mechanical ventilation is a medical intervention widely used in the ICU to treat critically ill patients, VAP is a form of pneumonia that can occur in these patients, particularly when mechanical ventilation is required for more than 48 h after tracheal intubation [38]. VAP develops following the entry of bacteria into the lower respiratory tract and is a condition that can be characterized by fever, lung infiltration, and altered white blood cell count [39,40]. The bacteriological etiology causing VAP can include innate oropharyngeal flora or those that have entered from the environment via aspiration and impaired ciliary action, while the duration of mechanical ventilation also largely contributes to the type of bacteria establishing the infection [38,39]. Consequently, these infections result in mortality in 13–25% of patients [39,40,41].

ET biofilm formation is considered one of the factors that contributes to VAP [42,43]. Contributing to ET biofilm production are pathogens, namely, *P. aeruginosa*, *A. baumannii*, and *S. aureus*. Biofilm formation on ET occurs early in intubated patients and becomes a reservoir for pathogens that can also rapidly colonize the lower respiratory airways [42,43,44]. Biofilm-producing *A. baumannii* colonize the respiratory tract for longer periods of time in comparison to non-biofilm forming isolates [45,46]; consequently, the heightened colonization duration increases patients’ risk of developing pneumonia. Biofilm quantification studies revealed that 75% of clinical isolates of *P. aeruginosa* from the ETs of patients with VAP were biofilm producers [47], which may also contribute to a higher and prolonged adherence capability, as reported with *A. baumannii*. Conversely, while Alonso et al. also report that most *P. aeruginosa* strains isolated from VAP patients are high biofilm producers in terms of biomass, their studies reveal that the virulence and pathogenesis of *P. aeruginosa* in VAP infections are not dependent on biofilm formation, and these VAP isolates were in fact less virulent in in vivo studies [47]. This study highlights the need for additional work that explores the significance of biofilm production and virulence, both in vivo and in vitro, and whether biofilm onset is dependent on various environmental cues and whether the biofilm has a role at specific points of the bacterial life cycle. This work may also assist in understanding the behavior of biofilm-producing bacteria relative to the condition of the host, their immune response, and the ET environment. Shedding light on this area are the findings by Fernandez-Barat et al. [45], who demonstrated that a weak in vitro biofilm-producing strain can in fact produce more biofilm in vivo, suggesting that the microenvironment in an ET may influence biofilm production through certain environmental cues.

When considering the “role” of biofilm production in VAP, it is essential to note that 70% of patients with VAP had identical pathogens isolated from both ET biofilm and tracheal secretions, reinforcing the notion that biofilms do provide a persistent reservoir for pathogenic bacteria [48]. Taking a step away from biofilm studies conducted in the ICU, it becomes apparent that the assessment of biofilm formation in the clinical setting lacks a general standard of assessment. SEM analyses of biofilm production vary between studies, as some studies consider whether a biofilm is present while other studies may examine the coverage. Without a gold standard for the microscopic analysis of biofilm production in the clinical setting, biofilm properties such as density, thickness, onset of production, and maturity will continue to be dismissed or not considered in the analysis [46,49]. Interestingly, lacking in this area of study are data, research, or clarity regarding why biofilm formation in some ICU patients occurs on indwelling devices, while in other patients, it does not occur. Such details may provide invaluable ways to further understand biofilm formation in ICUs.

Alongside VAP, urinary tract infections (UTIs) also contribute to healthcare-associated infections; of these, approximately 80% involve catheter-associated UTIs. The routine use of urinary stents and catheters are causes of UTIs due to the contamination of indwelling devices [50,51]. Similar to VAP, indwelling-device-related UTIs are associated with biofilm formation, in which the common contaminants include *S. epidermidis*, *E. faecalis,* and *P. aeruginosa*. Contrary to ET biofilms, only 2.3% of catheterized patients in the ICU with UTIs were associated with biofilm production [50,51]. When the same study examined biofilm formation in catheter UTIs outside of the ICU, biofilm production was associated with 73% of patients. The rationale proposed for the stark difference in biofilm production between UTI patients in the ICU and other wards includes the stricter hygiene protocols followed in the ICU and the duration of catheterization. Other studies have established a correlation between the duration of catheterization and the risk of developing biofilm-associated infections [51].

### 3.3. Association of Biofilm Production with Multidrug Resistance Among Isolates from the ICU

The biofilm network, aside from the surface-binding properties it brings, also serves as a mechanism for antibiotic resistance, the transfer of resistance plasmids, and intracellular communication [52]. It therefore becomes evident that, besides biofilms assisting in the adherence of bacterial communities to medical devices, biofilm-forming bacteria may be at a selective advantage against antibiotic resistance. The close cell-to-cell contact permitted through biofilm production may allow bacteria to transfer plasmid-containing antibiotic resistance genes to one another, consequently enhancing the spread of antibiotic resistance among bacterial pathogens in the ICU [53]. Evidence of biofilm-associated antibiotic-resistant pathogens isolated from the ICU is found in reports of biofilm-producing *P. aeruginosa*, which displayed more than 57% resistance to the aminoglycoside, fluoroquinolones, and β-lactam group of antibiotics. Conversely, in the same survey, 20% of non-biofilm-producing *P. aeruginosa* were resistant to the same groups of antibiotics [6]. Similarly, *A. baumannii* isolates from the same ICU ward displayed higher levels (73%) of resistance to the aminoglycoside, fluoroquinolone, and β-lactam group of antibiotics, in comparison to non-biofilm producers, which showed lower levels of resistance (25%) [6]. Although this study was able to reiterate that there is a relationship between biofilm-producing bacteria and higher levels of multidrug-resistance patterns, it is important to acknowledge that the isolates were collected from infection sites and not from indwelling medical-device-associated biofilms. Comparatively, in another study, biofilm-producing clinical isolates of *A. baumannii* showed greater resistance to ampicillin–sulbactam, amikacin, ciprofloxacin, and ceftazidime [7]. Yet, in the same study, lower resistance to imipenem and piperacillin was observed in biofilm formers of *A. baumannii*, which was believed to be a result of both antibiotics being able to penetrate through the biofilm.

If attention is re-directed to studies that investigated antibiotic resistance amongst in-dwelling-device-related infections, the same correlation between antibiotic resistance and biofilm production seems to be apparent [54,55]. For instance, biofilm-producing bacterial isolates from catheter-related infections and ventilator-associated pneumonia were multidrug-resistant [56,57,58]. Prevalent bacteria among these isolates were *P. aeruginosa*, *A. baumanii*, and *Staphylococcus* spp. [56,57,58,59]. Resistance to all antibiotics tested was reported, except for vancomycin among gram-positive strains and imipenem among gram-negative strains [53]. In a separate study, the biofilm community that colonized the oropharynx and endotracheal tubing of ICU patients consisted predominately of MDR *P. aeruginosa*, which showed resistance to cefepime, ceftazidime, gentamicin, and ofloxacin, while the MDR *A. baumannii* isolates displayed resistance to trimethoprim/sulfamethoxazole, cefotaxime, and amikacin [4]. Studies that drew comparisons between the frequency of MDR strains that are biofilm producers and MDR strains that are non-biofilm producers were able to demonstrate that biofilm formation is higher in MDR strains [6,53,60,61,62,63]. The persistence of these of organisms is therefore heightened through resistance to antimicrobial therapies and colonization capacities through biofilm formation.

The incidence of antibiotic-resistant biofilm producers appears not be confined to one bacterial type but rather is present across various species that are most commonly reported in device-related infections in the ICU. Is it therefore likely that these strains that are capable of forming biofilms are selected under antibiotic pressure or vice versa? To gain insight into this area, it is important to consider the molecular mechanisms associated with biofilm formation, the MDR phenotype, and other virulence factors (refer to Section 4). Additionally, it becomes crucial to assess and understand the various resistance patterns of organisms specific to the ICU for the optimum management and treatment of such infections (refer to Section 3.4).

### 3.4. Treatment of Biofilm Producing MDR Strains

Treating chronic biofilm formation while simultaneously overcoming antibiotic resistance continues to be one of the most problematic issues for hospitalized patients. Observations that the density of biofilm in resistant strains can be greater than in susceptible ones [60,64] adds to the complexity of treatment plans. Also, the detection of the same pathogen within an ET biofilm as in the lung of a patient reinforces the claim that the biofilm acts as a reservoir for the persistence of pathogenic bacteria when treatment is not successful [10,60]. Additionally, biofilm-encased bacteria may also require antibiotic concentrations up to 1000 times higher than planktonic bacteria in order to be eliminated [37].

When the antibiotic resistance profile of biofilm-encased pathogens isolated from the oropharynx and ET tubing are examined, the risk of developing VAP in ICU patients becomes evident. Studies have found MDR *P. aeruginosa* strains with resistance to cefepime, ceftazidime, gentamicin, and ofloxacin, while MDR *A. baumannii* strains had high resistance levels to trimethoprim/sulfamethoxazole, cefotaxime, ciprofloxacin, and amikacin [4]. An underlying cause of the resistance conferred is the production of ESBL and/or AmpC amongst these strains [4,64]. It becomes evident that the treatment plan is dependent on the antibiotic resistance profile and the genetic predisposition of these strains. Here, one must also acknowledge that resistance mechanisms, whether innate or acquired, can exist simultaneously, in turn enhancing the ineffectiveness of conventional antibiotic treatments. Alternative effective treatments on such strains include treatment with imipenem, polymyxin B, and colistin [4]. Combination treatment with colistin and polymyxin B has also been suggested as an effective treatment option for MRSA and *P. aeruginosa* strains [65], while fosfomycin treatment of MDR *P. aeruginosa* strains has also been suggested as an effective treatment option [66].

MRSAs have been isolated from biofilms formed over the ET cuff in the ICU and are consequently one of the major causes of ICU-acquired pneumonia [67]. ET biofilm formation has been recognized as one of the factors that can lead to VAP and/or to the relapse of VAP. A comparative study that investigated the treatment of MRSA respiratory infection with linezolid in mechanically ventilated ICU patients found that the presence and load of ET biofilm and MRSA, respectively, were significantly lowered. Systemic linezolid treatment limited biofilm production and ET MRSA at a 67% higher rate when compared to treatment with vancomycin [45]. The strength of such a treatment method is the simultaneous reduction in biofilm production while inhibiting bacterial growth. Conversely, to date, there is limited information regarding how antimicrobials can affect biofilm production.

As the challenges in the treatment of ICU biofilm-induced infections continue, and the available clinical solution for biofilm-encased antibiotic-resistant bacteria may not always suffice, attention to the materials used in-dwelling medical devices has been considered as a possible preventive strategy to reduce biofilm production [68,69,70]. Concepts have been developed to protect ET surfaces against biofilm colonization through the application of antimicrobials on the ET surface that are released by diffusion [68,70]. The benefits of the bactericidal coating of the ET alone, however, appears to be temporary, and conversely, studies that apply the technique of mucous shaving to bactericidal-coated ETs were able to prevent bacterial growth and the accumulation of secretions within the ET following mechanical ventilation for 72 h [66].

Silver-coated ETs have also been proven to be effective in inhibiting the growth of biofilm-encapsulated bacteria and have undergone clinical trials to demonstrate their beneficial effects [49]. Thiol groups found in the extracellular matrix are oxidized through the binding of the silver ions and inactivating the respiratory chain proteins, leading to delayed ET colonization [66]. Silicon-coated and noble-metal-coated PVC ETs have also been associated with reduced biofilm formation compared with standard uncoated PVC tubing [46]. To further tackle the initial 24 h colonization within the ET, some studies have explored the method of antimicrobial photodynamic therapy (aPDT). This method of in vitro studies was demonstrated to be successful in significantly reducing ET biofilms in a single treatment. The method is noninvasive and relies on light to activate light-sensitive photosensitizers to destroy microbial pathogens. Photoactive dyes are applied to a colonized ET and, once absorbed by the pathogens, a specific wavelength of light is applied, and the pathogens can be eliminated, in turn reducing biofilm formation and enhancing biofilm detachment from ET walls [71,72,73].

## 4. Unraveling the Molecular Genetics of Biofilm Production and Antimicrobial Resistance in the ICU

Collectively, bacteria will exhibit an array of “survival tools” including biofilm formation, quorum sensing, and horizontal gene transfer in order to adapt, grow, and survive in sub-optimum conditions [71,74,75]. These genetic properties have allowed bacteria to acquire resistance to multiple drugs, reside within a biofilm, permit high levels of colonization, and consequently further the dissemination of bacteria in the hospital setting. In-dwelling medical devices/implants among ICU patients are considered one of multiple factors that provide a platform for biofilm-forming bacteria to flourish and persist [76].

Often, the added challenge to treating infections among ICU patients is that microbial confirmation can take between 48 and 72 h; this time span can provide the sufficient time required for biofilms to form on in-dwelling medical devices [23,33,34,40]. Little is known about how antimicrobials affect biofilm formation on in-dwelling medical devices and whether the molecular mechanisms involved in biofilm production and antibiotic resistance simultaneously exist, thus conferring both combined properties continuously. It is also important to recognize that the molecular mechanisms and environmental cues responsible for the cascade of events that lead to biofilm production and resistance may differ between bacteria.

When considering the literature that concentrates on biofilm-related ICU infections, there are limited studies that have aimed to selectively investigate or identify therapies capable of inhibiting or preventing biofilm growth following molecular genetic investigations [67,77,78,79]. There is, however, a more apparent presence of studies that investigate the antibiotic resistance mechanisms specific to pathogens relevant to ICU and not necessarily associated with biofilms [10,13,20,59,80,81,82].

Biofilm-forming *S. aureus* and *S. epidermis* are both recoverable from infections associated with indwelling devices. The gene products of the operon referred to as *icaABCD* are responsible for polysaccharide intracellular adhesion, which mediates cell-to-cell adhesion [83,84]. Concurrently, biofilm production is achieved by staphylococci, as in other bacteria, through quorum sensing. Through the production and release of quorum-sensing signaling molecules, “autoinducers”, gene expression is regulated in response to fluctuations in cell density [85]. Since bacterial cell-to-cell communication is crucial in biofilm production [30,86], attempts have been made to inhibit the cell-to-cell communication and hence biofilm formation using a quorum-sensing inhibitor (RNAIII-inhibiting peptide). Reports of hamamelitannin, a natural polyphenol extract that acts as an analog of RNAIII-inhibiting peptide, plays a significant role in preventing biofilm formation. As a quorum-sensing blocker, it suppresses biofilm formation without killing bacteria. This alternative treatment concurrently minimizes the development of resistant strains by removing the selective pressure [87]. Although the logistics were not discussed, the authors suggested the lining of indwelling medical devices with an analog of a quorum-sensing inhibitor to prevent biofilm accumulation as a preventive approach.

Biofilm-producing *A. baumanii* and *P. aeruginosa* collected from various ICU units displayed the presence of essential biofilm-producing genes, *bap* (biofilm-associated protein) and *rhlI *(quorum sensor auto-inducer) [88], regardless of whether the strains were classified as high or moderate biofilm producers. Other studies that compared *rhlI* mutants of *P. aeruginosa* with wild-type strains demonstrated that biofilm production was reduced by 70% [86]. A more complete understanding of the genetics between high, moderate, and low biofilm producers from the ICU may provide insight into the regulatory cascade and whether differences between the microenvironment that strains are isolated from influence gene expression and in turn the levels of biofilm produced. To coincide with such investigations, extracellular polymeric substances (EPSs) should be considered from a molecular perspective to determine which EPS genes are expressed among strains isolated from various indwelling devices. For example, it is known that *P. aeruginosa* biofilms can be composed of three different EPSs. These points need to be addressed in the ICU context in order to prevent or reduce biofilm colonization of the ICU and indwelling medical devices.

The ICU provides a unique environment for creating and disseminating antibiotic-resistant organisms. As already described in Section 3.4, the selection pressure in the ICU is highest due to the increased use of antibiotics to treat already critically ill patients. The vulnerability of ICU patients to infections is due to their delayed immune response, combined with the use of invasive indwelling medical devices. Included in this scenario of biofilm formation, the “perform storm” becomes apparent with the emergence of exceptional antibiotic-resistant bacteria that are able to survive for long periods. Pathogens termed resistant are inhibited or killed by certain concentrations of antibiotics suitable for use in patients. Clinical resistance requires the consideration of multiple points, including the immune status of the patient, the site of infection, and the infecting organism [59].

The mechanisms adopted by bacteria to confer resistance have been well documented and explored in various bacteria [11,57,76,83,89]. To remain in the scope of this review, the MDR pathogens responsible for ICU acquired infections and considered as highly important ICU pathogens will be considered. *A. baumannii*, *P. aeruginosa*, *Klebsiella,* and *Staphylococci* have innate resistance to various antibiotics and antiseptics [52,83]. Innate resistance mechanisms may involve efflux pumps, low permeability of the outer membrane, bypass pathways, and enzymatic inactivation or modification [74]. Limiting the efficiency of antibiotics against such bacteria can be a consequence of these various mechanisms existing simultaneously, hence conferring resistance to many antibiotics. Alternatively, acquired resistance, as the name suggests, is resistance acquired through the acquisition of antibiotic resistance genes or through mutational events in genes encoding binding proteins, efflux pumps, chromosomal enzymes, and porins [11,57,73,76,83]. The biofilm environment is thought to facilitate the transfer of resistance genes between strains via plasmids and/or mobile genetic elements (e.g., transposons, integrons), which then integrate into the bacterial genome [1,34,76]. This synergetic relationship between biofilms and antibiotic resistance may be a consequence of the close proximity cells remain in for a prolonged period of time, permitting the acquisition of genes [17,31,34]. Additionally, bacteria are able to undergo genomic alterations that are mediated by transposons or through horizontal gene transfer; consequently, with gene rearrangement, it is believed that a single enzyme may have the capacity to exhibit multiple functions. If antibiotic resistance is considered, a single gene product may be able to nullify the bactericidal effect of different antibiotics, which may pose an additional challenge during the treatment of infections [90].

A substantial body of work has established that biofilm formation appears to occur in stages, from the initial attachment of cells to a surface and adhesion through EPS production to the development of a mature biofilm and then the active release of planktonic cells to the surrounding environment for colonization to the other sites [1,2,61]. It is also important to acknowledge that mature biofilms may contain different bacterial species; it becomes apparent how the biofilm space allows for genetic transfer and hence enriches the environmental adaptability of bacteria. Additionally, there is a large array of studies and continuing investigations into antibiotic-resistant phenotypes and mechanisms that allow MDR bacteria to dominate the ICU and the general hospital setting [91,92]. A more complete understanding of the interplay between biofilm production and antibiotic resistance is required. Although this review has purposely been biased toward studies that have taken place in ICUs, a recent study on *A. baumannii* samples not collected from the ICU provides grounds for similar investigations for comparative purposes to occur on ICU medical-device-dwelling biofilm producers. Through this investigation, antibiotic resistance, related biofilm genes, and biofilm production all shared a correlation with certain antibiotics, enhancing biofilm production [93].

Evidently, as discussed, biofilm growth is associated with quorum-sensing regulated mechanisms, while increased levels of gene transfer exhibit antimicrobial tolerance [1,32,37,86,94]. However, little is still known about how antimicrobials affect biofilm formation on in-dwelling medical devices. Possibly, the many facets involved with biofilm production combined with the challenges the ICU environment presents, may hinders the ability to identify critical key elements which induce biofilm colonization of in-dwelling devices and whether the onset of biofilms is equivalent in all chronic infections.

## 5. Future Directions: Alternatives to Traditional Methods; Is There Any Good News?

### 5.1. Bacteriophage Therapy

With a plateau in novel antibiotics, alternative therapeutics are required to combat antimicrobial resistance and persistent nosocomial bacterial infections. A potential substitute to traditional biofilm treatment is the use of bacteriophages and phage therapy. Bacteriophages are viruses that infect and replicate within bacterial hosts. As one of the most abundant entities on Earth (~10^31^) [95], phages are ubiquitous in nature and can be classified as either virulent (lytic) or temperate (lysogenic) depending on their biological cycle. Although first discovered in the early 20th century, phages and their use as therapeutic agents were neglected following the introduction of clinically successful antibiotics. As the efficacy of current antibiotics is declining, these viruses have recaptured attention as novel methods to combat bacterial-driven conditions. Previous research has confirmed that resistance mutations against common antibiotics do not hinder phage infection. Bacteriolytic activity has been observed in multiple MDR species [96]. Recently, phage therapy has emerged as a promising approach to combat persistent nosocomial infections. While bacteriophage therapy has been extensively studied in the context of “ESKAPE” organisms, viral infection of biofilms presents distinct challenges compared to planktonic cells. Diffusion and absorption within these biofilm matrices pose significant obstacles for phages and can be influenced by factors such as biofilm age, density, and molecular composition [97]. These complexities may impact the effectiveness of bacteriophages in treating chronic biofilm infections.

The analysis of 15 MDR *A. baumannii* isolates revealed an 87% reduction in biofilm contents following treatment with lytic Myoviridae bacteriophage [98]. Although promising, this phage treatment did not completely eliminate the entire biofilm matrix in some isolates [98], underscoring the need for synergistic combination therapies. Current research has shifted to identify favorable biofilm treatment combinations, including phage cocktail therapies, phages in combination with effective antibiotics, and the use of phage-derived enzymes including endolysins. Further, the in vitro treatment of a biofilm-producing *S. aureus* strain highlighted increased antibacterial activity when lytic bacteriophage treatment preceded the use of antibiotics vancomycin and cefazolin [99]. Similar results were shown in vitro using ET-associated *P. aeruginosa* biofilms, with a decrease in biomass observed when phage treatment was followed by gentamicin exposure 6 h post phage treatment [100]. These findings highlight the complexity of biofilm elimination. Although phage therapy provides a promising alternative to traditional methods of biofilm elimination, further insights into dose optimization, treatment duration, and optimal phage/antibiotic combinations are required prior to treatment in clinical settings.

### 5.2. Artificial Intelligence a Potential Way to Decipher Biofilm Production

As artificial intelligence (AI) applications continue to evolve and become integrated into multiple aspects of healthcare, the use of AI has also been considered for the detection and treatment of biofilms and biofilm-associated infections. The automated detection of biofilms has recently been described in the field of medical diagnosis. A convolutional neural network (CNN)-based biofilm detection system has been proposed for the rapid and accurate detection of biofilms in the field of rhinocytology. The developed algorithms were reported to analyze the chromatic and morphological characteristics of biofilms with an accuracy of 98% [101]. Similarly, the automated detection of dental biofilms via visual segmentation based on deep learning techniques [102], as well as the quantification of bacterial adhesion and biofilm formation on dental materials, have been reported to have high sensitivity and specificity [103]. Machine-learning-aided cocktail assays for reliable biofilm detection have also been suggested for the prompt diagnosis of biofilm infections [104].

Most studies exploring biofilm mechanisms rely on omics studies, such as transcriptomics and proteomics, which can uncover new genetic and protein targets for new anti-biofilm agents. In silico screening can also be used to search for molecules in large databases that may bind and modulate these targets. The newer approach is to use machine learning, in which developed algorithms are employed to predict the anti-biofilm activity of molecules [105]. To address the growing concern of antibiotic-resistant pathogens associated with biofilm infections worldwide, a number of studies have focused on the new approach for the discovery and identification of molecules with anti-biofilm activity. These studies have used machine learning algorithms for the prediction of chemical components of organic compounds, for example, in essential oils (EOs), which have higher biofilm inhibition potential. As a strategy to mitigate bacterial virulence, hamper bacterial cell adhesion, and reduce biofilm formation, machine learning has been applied in the development of classification methods to predict possible antibiofilm action for each chemical component of EOs under investigation. Using this strategy, the authors were able to detect chemical components responsible for the inhibition of bacterial biofilms formed by *S. aureus*, *S. epidermidis* and *P. aeruginosa* [106]. More recently, the Biofilm-I platform and Molib tool have been developed for the prediction of biofilm inhibitors using the quantitative structure–activity relationship of small molecules and used against important drug-resistant pathogens, including *E. coli*, *P. aeruginosa*, *S. aureus,* and *Candida albicans* [107,108,109].

**Figure 1:**
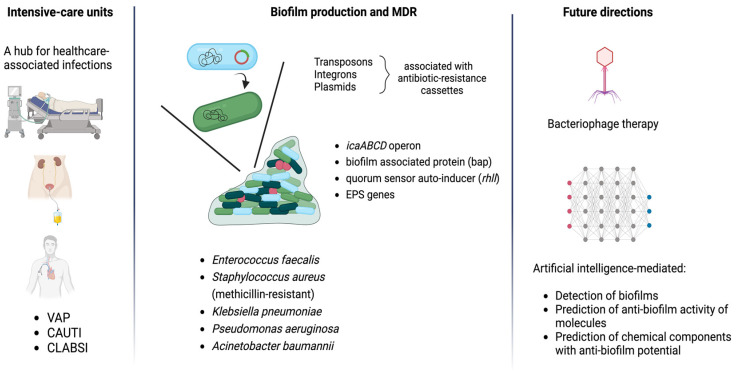
A schematic diagram representing how ICUs harbor biofilm-producing MDR bacteria while highlighting the complex interplay of biofilm production and antibiotic resistance. Current research has shifted to identifying favorable biofilm treatment combinations, including phage cocktail therapies in combination with effective antibiotics, while developed algorithms are employed to predict anti-biofilm activity of molecules (image created using image database Biorender).

Figure 1 illustrates a schematic diagram that summarizes how the ICU is associated with biofilm-producing multi-drug-resistant bacteria and how future methods may assist in the management of ICU-related biofilm production.

## 6. Conclusions

The presence of biofilm-producing bacteria in ICUs has been and continues to be a universal phenomenon. Perhaps the presence of biofilm-producing bacteria in the ICU is inevitable, as the ICU environment appears to provide adequate selective measures for biofilm formation, allowing bacterial cells to form a resistant community and survive transient eradication methods. Currently, antibiotic combination therapies continue to be the treatment of choice, and although there has been a noticeable positive shift in the adherence to infection control measures and antimicrobial stewardship in the ICU over the years, infection control challenges continue regarding biofilm-encased antibiotic-resistant bacteria.

Some progress has been made in developing future therapies to reduce, monitor, and manage the occurrence of biofilm formation. Current research has shifted to identifying favorable biofilm treatment combinations, including phage cocktail therapies, phages in combination with effective antibiotics, and the use of phage-derived enzymes, including endolysins, while machine-learning-aided cocktail assays have been developed for reliable biofilm detection and the prompt diagnosis of biofilm infections. Such future therapies in combination with effective antibiotic treatment may alleviate ICU biofilm-related infections.

For future therapies to take hold, there may need to be a deeper understanding of the timeline of biofilm formation on surfaces and indwelling medical devices. There also needs to be a better understanding of how individual cells within the biofilm matrix may differ with regard to EPS production, which may in turn influence the density of the overall biofilm formed. Furthermore, by better understanding the molecular regulatory system involved in biofilm production, both at the aggregate and the individual cell level, then, perhaps, future therapies combined with antibiotic treatment can be more strategically implemented to manage biofilm production in the ICU.

## Data Availability

All articles and data sources reviewed in this review are publicly available online. Readers can access the full-text articles and associated data through the respective journal websites or repositories where they were originally published or hosted.
